# Management of severe acute encephalopathy in the ICU: an expert consensus statement from the french society of intensive care medicine

**DOI:** 10.1186/s13613-025-01436-0

**Published:** 2025-03-21

**Authors:** Romain Sonneville, Eric Azabou, Pierre Bailly, Sarah Benghanem, Gilles De Almeida Cardoso, Pierre Claquin, David Cortier, Augustin Gaudemer, Bertrand Hermann, Pierre Jaquet, Virginie Lambrecq, Camille Legouy, Stéphane Legriel, Thomas Rambaud, Benjamin Rohaut, Benjamine Sarton, Stein Silva, Tarek Sharshar, Fabio Silvio Taccone, Dominique Vodovar, Nicolas Weiss, Charles Cerf

**Affiliations:** 1 Médecine intensive reanimation, Hôpital Bichat - Claude Bernard, 46 Rue Henri Huchard, 75877 Paris Cedex, France; 2 Université Paris Cité, IAME, INSERM, UMR 1137, 75018 Paris, France; 3 Clinical Neurophysiology and Neuromodulation Unit, Departments of Physiology and Critical Care Medicine, Inserm UMR 1173, Infection and Inflammation (2I), Raymond Poincaré Hospital, Assistance Publique- Hôpitaux de Paris, University of Versailles Saint-Quentin en Yvelines (UVSQ), Paris-Saclay University, Garches, Paris, France; 4 Médecine intensive reanimation, CHU de Brest, Brest, France; 5 Médecine intensive reanimation, Hôpital Cochin, Paris, France; 6 Service de reanimation medico-chirurgicale Hôpital Foch, Suresnes, France; 7 Imagerie medicale, Hôpital Bichat - Claude Bernard, Paris, France; 8 Médecine intensive reanimation, Hôpital Européen Georges Pompidou, Paris, France; 9 Médecine intensive reanimation, Hôpital Delafontaine, Saint Denis, France; 10 DMU Neurosciences, Département de Neurophysiologie Clinique, Paris Brain Institute – ICM, Inserm U1127, Sorbonne Université, APHP, Hôpital Pitié-Salpêtrière, CNRS-UMR7225, Paris, France; 11 Anesthesia and intensive care department, Pole Neuro, GHU Paris Psychiatrie et Neurosciences, Sainte Anne Hospital, Paris, France; 12 INSERM U1266, Institute of Psychiatry and Neurosciences of Paris, Université Paris Cité, Paris, France; 13 Médecine intensive reanimation, CH Mignot, Le Chesnay, France; 14 DMU Neurosciences - Neuro ICU, PICNIC-Lab, Sorbonne Université, APHP, Hôpital de la Pitié Salpêtrière, Paris Brain Institute - ICM, Inserm, CNRS, Paris, France; 15 Service de reanimation Polyvalente Hôpital Purpan, CHU de Toulouse, Toulouse, France; 16 Service des Soins intensifs, Hôpital Universitaire de Bruxelles (HUB), Université Libre de Bruxelles (ULB), Brussels, Belgique; 17 Centre Antipoison de Paris, AP-HP, Hôpital Fernand Widal, 75010 Paris, France; 18 Inserm, Optimisation Thérapeutique en Neuropsychopharmacologie, Université Paris Cité, 75006 Paris, France; 19 UFR de médecine, Université Paris-Cité, 75010 Paris, France

## Abstract

**Introduction:**

Acute encephalopathy in the ICU poses significant diagnostic, therapeutic, and prognostic challenges. Standardized expert guidelines on acute encephalopathy are needed to improve diagnostic methods, therapeutic decisions, and prognostication.

**Methods:**

The experts conducted a review of the literature, analysed it according to the GRADE (Grading of Recommendation, Assessment, Development and Evaluation) methodology and made proposals for guidelines, which were rated by other experts. Only expert opinions with strong agreement were selected.

**Results:**

The synthesis of expert work and the application of the GRADE method resulted in 39 recommendations. Among the 39 formalized recommendations, 1 had a high level of evidence (GRADE 1 +) and 10 had a low level of evidence (GRADE 2 + or 2-). These recommendations describe indication for ICU admission, use of clinical scores and EEG for diagnosis, detection of complications, and prognostication. The remaining 28 recommendations were based on expert consensus. These recomandations describe common indications for blood and CSF studies, neuroimaging, use of neuromonitoring, and provide guidelines for management in the acute phase.

**Conclusion:**

This expert consensus statement aims to provide a structured framework to enhance the consistency and quality of care for ICU patients presenting with acute encephalopathy. By integrating high-quality evidence with expert opinion, it offers a pragmatic approach to addressing the complex nature of acute encephalopathy in the ICU, promoting best practices in patient care and facilitating future research in the field.

## Introduction

Acute encephalopathy is a syndrome characterized by a rapidly developing (typically hours to days, less than 4 weeks) pathobiological brain process which is expressed clinically either as delirium or coma, both representing a change from baseline cognitive status. Additional clinical features may be observed depending on etiologies, including seizures, movement disorders, and dysautonomia [1]. Acute encephalopathy is commonly associated with acute systemic processes (i.e. sepsis, metabolic derangements/disorders, intoxications, or withdrawal syndromes). Less frequently, it may be an indicator for an acute cerebral disease of infectious, inflammatory, metabolic, or vascular origin. The term acute encephalopathy is not recommended as a descriptor of clinical features that can be observed at the bedside. Experts recommend the term subsyndromal delirium for acute cognitive changes that are compatible with delirium, but do not fulfil all DSM-5 delirium criteria [1], the term delirium for a clinical state defined according to the criteria of the DSM-5 [2], and coma for a state of severely depressed responsiveness defined using diagnostic systems such as the Glasgow Coma Score (GCS) [3] or the Full Outline of UnResponsiveness (FOUR) score [4].

Acute encephalopathy entails a considerable short-term risk to life and may result in prolonged hospital stays, persistent neurological sequelae and altered quality of life in survivors, irrespective of clinical presentation.

We propose recommandations for the diagnosis, management, and prognosis among patients with severe acute encephalopathy (SAE), requiring care in the intensive care unit (ICU). We specifically exclude from these recommandations encephalopathies arising from head trauma, acute cerebrovascular pathologies (i.e. subarachnoid hemorrhage, intracerebral hemorrhage), and successfully resuscitated cardiac arrests. Of note, these distinct pathologies have been subject to previous separate recommendations [5–8].

## Methods

These recommendations are the result of the collaborative efforts of an expert panel convened by the SRLF (French Intensive Care Society). The group’s agenda was predetermined, beginning with the identification of key questions by the organizing committee in consultation with coordinators. Subsequently, experts were assigned to address each question. The questions were framed using the PICO format (Patient Intervention Comparison Outcome) following an initial expert group meeting.

A level of evidence was defined for each publication cited as a function of the study design. This level of evidence could be revised by taking into account the methodological quality of the study. A global level of evidence was determined for each endpoint by considering the levels of evidence of each publication, the consistency of the results between the various studies, the direct or indirect nature of the evidence, and the cost analysis (Table 1).

**Table 1:**
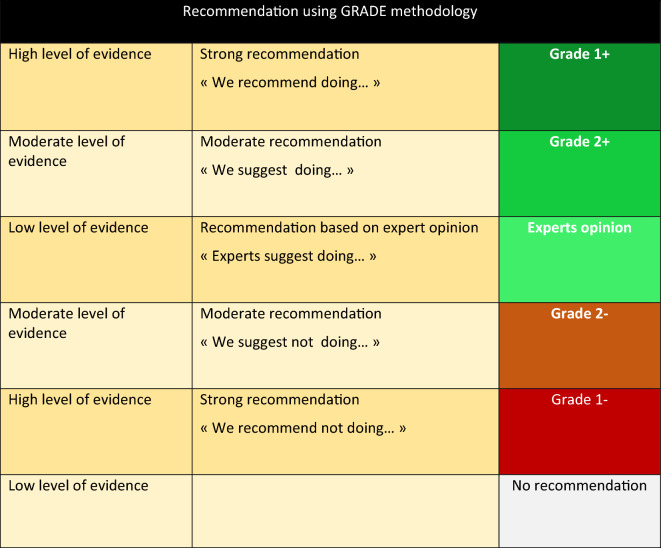
Recommendation with GRADE methodology

A “strong” overall level of evidence led to the formulation of a “strong” recommendation (must do, must not do … GRADE 1 + or 1-). An overall level of evidence categorized as “moderate,” “low,” or “very low” resulted in an “optional” recommendation (probably should do, probably should not do, … GRADE 2 + or 2-). In cases where literature was absent or insufficient, the question could be addressed with an expert opinion (experts propose …).

Proposed recommendations were presented and discussed one by one. The purpose of this process was not to inevitably reach a unique, convergent expert consensus on all of the proposals, but to define points of concordance, divergence or indecision. Each recommendation was then evaluated by each of the experts, who provided an individual score using a scale ranging from 1 (complete disagreement) to 9 (complete agreement). The collective score was established according to a GRADE grid methodology. To obtain a strong agreement, 70% of experts had to agree with the recommendation. In the absence of a strong consensus, the recommendations were reformulated and rescored in order to reach a consensus. Only expert opinions that obtained a strong agreement were fnally adopted.

Four fields of recommandations were defined: (1) Diagnostic approach; (2) Indications, and methods of neuromonitoring; (3) Prognostication of awakening and neurologic sequelae; and (4) Management (excluding etiological treatment). A literature search (2000–2023) limited to adult studies was conducted using MEDLINE via PubMed and Cochrane databases. Publications were included in the analysis if they were in English or French. The analysis focused on recent data in order of preference, from meta-analyses and randomized trials to observational studies.

## Results

The synthesis of expert work and the application of the GRADE method resulted in 39 recommendations. Among the 39 formalized recommendations, 1 had a high level of evidence (GRADE 1 +) and 10 had a low level of evidence (GRADE 2 + or 2-). For 28 recommendations, the GRADE method could not be applied, leading to expert opinions. After two rounds of rating and amendments, a strong agreement was reached for all 39 recommendations.

**FIELD 1: Diagnostic approach**

***Question 1.1: In a patient with SAE, what are the initial phase severity criteria that necessitate admission to the intensive care unit (ICU)?***

**R 1.1.1: Apart from rapidly reversible causes, patients with SAE exhibiting coma features should probably be hospitalized in an ICU.**

GRADE 2 + / STRONG AGREEMENT.

**R 1.1.2: Apart from rapidly reversible causes, patients with SAE exhibiting respiratory control abnormalities, upper airway protection issues, or concurrent organ failure should probably require hospitalization in an ICU.**

GRADE 2 + / STRONG AGREEMENT.

**R 1.1.3: In patients with SAE, experts suggest ICU admission for those exhibiting dysautonomia.**

EXPERT OPINION/ STRONG AGREEMENT

*Rationale* The decision to admit a patient with SAE to the ICU depends mainly on neurological severity and its possible respiratory consequences. The type and intensity of associated neurological symptoms, the evolving potential of the underlying mechanism or the etiology must be considered, as well as the means required for symptomatic or etiological management [2]. The criteria for ICU admission for a patient with SAE are mainly based on low level of evidence studies and expert recommendations. Coma is an undisputed criterion for ICU admission, regardless of its cause [3, 4]. The presence of delirium is not sufficient to justify ICU admission, as no study has assessed to what extent a delirium will progress to a coma or lead to respiratory failure. Patients who are unable to protect their upper airways or who have respiratory control abnormalities must be hospitalized in ICU. ICU admission should be discussed in the presence of seizures/status epilepticus and in the presence of non-neurological organ failure. Special attention should be paid to patients presenting with hyperactive delirium [2]. For example, patients with delirium tremens complicating alcohol withdrawal syndrome usually require close monitoring, intravenous medications, and sometimes physical restraints, which cannot be done safely outside the ICU environment.

***Question 1.2: In a patient with SAE, what type of cerebral imaging is necessary for etiological assessment?***

**R 1.2.1: In the absence of an obvious cause, experts suggest performing a non-contrast head computed tomography (CT) in the acute phase, to exclude intracranial hemorrhage or cerebral infarction.**

EXPERT OPINION

**R 1.2.2: In the presence of coma or signs of brainstem involvement, experts suggest conducting both a head CT and an angio-CT of the Willis polygon to rule out basilar artery occlusion.**

EXPERT OPINION

**Fig. 1:**
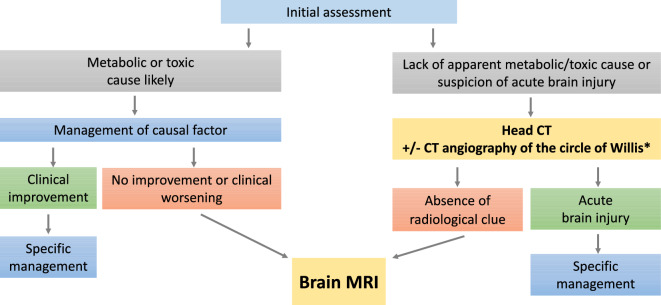
Decision algorithm for neuroimaging in the initial phase of severe acute encephalopathy. *In the presence of coma or signs of brainstem involvement, experts suggest conducting both a cranial CT scan and an angio-CT of the circle of Willis polygon to rule out basilar artery occlusion

**R 1.2.3: Experts suggest obtaining a brain MRI when faced with a persistent SAE without clear clinical, biological, or radiographic etiological clues, or when there is no improvement despite an identified cause (**Fig. 1**).**

EXPERT OPINION

**R 1.2.4: When an MRI is performed, experts suggest routinely including a diffusion-weighted sequence to detect specific abnormalities (of infectious, metabolic, and toxic origin) (**Table 2**).**

**Table 2:** MRI clues to the diagnosis of severe acute encephalopathy

Etiology	Topography	Diffusion-weighted imaging (DWI)	Hemorrhage	Enhancement	Comments
*Herpetic encephalitis*	Hyperintensity in cortico-subcortical region on DWI and T2 FLAIR sequences Asymmetric or even unilateral involvement in 21–94% Involvement: - temporal: 84–100% - insular: 70% - fronto-basal: 41–68%	Restriction 29–65%	5–10%	12–70%	In encephalitis cases with temporal involvement, herpetic encephalitis more frequently exhibits isolated temporal involvement (30%) compared to other types of encephalitis with temporal involvement (particularly limbic encephalopathy)
*Hepatic encephalopathy*	Hyperintensity on T2 FLAIR and DWI Bilateral and symmetrical involvement: - cortical: 30–100% - thalamus: 25–100% - basal ganglia: 0–100% - white matter: 0–80%	Restriction			Involvement of the insula Relative preservation of the motor cortex on DWI Association with other signs of chronic hepatic encephalopathy (T1 hyperintensity in the pallidum)
*Hypoglycemia*	Hyperintensity on T2 FLAIR and DWI Bilateral and symmetrical involvement: - white matter, particularly the posterior limbs of the internal capsules: 36–82% - cortical: 71–73% - hippocampus: 29–36% - gray nuclei: 8–47%				MRI may be normal in cases of very acute severe hypoglycemia
*Wernicke’s encephalopathy*	Hyperintensity on T2 FLAIR and DWI Bilateral and symmetrical involvement: - thalamus: 46–94% - peri-aqueductal area: 40–100% - mamillary bodies: 18–100% - cortical: 17–33% - posterior fossa: 3–27%				The involvement may vary depending on the patient's alcohol status, with more atypical forms in non-alcoholic patients (more frequent involvement of the cerebellum, cranial nerve nuclei, and mamillary bodies)
*Posterior reversible encephalopathy syndrome (PRES)*	Hyperintensity in cortico-subcortical FLAIR sequences Bilateral involvement, rarely unilateral (1–13%): - parieto-occipital: 55–100% - frontal: 51–89% - cerebellum: 22–53%	Restriction (foci within a vasogenic edema zone): 8–60%	17–33% (hematomas 11–19%)		"Reversible" nature most common but inconsistent (Ni 2011: 3 patients out of 18 with irreversible lesions)
*Carbon monoxide poisonning*	Hyperintensity on FLAIR and DWI Bilateral and symmetrical involvement: - globus pallidus: 20–60% - other basal ganglia or thalamus nuclei: 4% - cortex: 9% - white matter: 7%	Restriction 90–100%			

EXPERT OPINION

*Rationale* The prevalence of abnormalities on CT scans is approximately 10% [9], and likely lower when a toxic or metabolic factor is present [10]. Lesions identified on CT are primarily ischemic or hemorrhagic [11]. Basilar artery occlusion accounts for approximately 10% of non-traumatic causes of coma, justifying CT-angiography [12]. A delayed contrast-enhanced CT scan probably does not provide additional diagnostic information [13]. MRI likely allows better detection of ischemia in acute encephalopathy [14], but the overall benefit of MRI is not proven [15]. Lesions observed in sepsis-associated encephalopathy are mostly small ischemic lesions and FLAIR hyperintensities [16, 17]. The diffusion-weighted sequence increases MRI sensitivity in toxic, infectious, autoimmune and metabolic pathologies, and reversible posterior leukoencephalopathy syndrome (PRES) [18–21]. Immunocompromised patients represent a subset of patients at high risk for developing intracranial complications, including stroke, metabolic/toxic encephalopathy, and brain infections. Brain MRI might be recommended as first-line imaging investigation in these patients to rule out specific complications.

***Question 1.3: In a patient with SAE, when is it necessary to urgently perform an electroencephalography (EEG), and what abnormalities should be investigated for etiological purposes?***

**R 1.3.1: In patients with SAE, a standard EEG should probably be conducted urgently to rule out non-convulsive seizures or nonconvulsive status epilepticus.**

GRADE 2 + / STRONG AGREEMENT.

**R 1.3.2: Experts suggest systematically assessing EEG reactivity to stimulation and presence of abnormalities suggestive of a specific etiology (**Table 3**).**

**Table 3:** EEG abnormalities and etiological diagnosis of severe acute encephalopathy

EEG abnormalities found in SAE	
Slow background activity Anterior slow waves, FIRDA (Frontal Intermittent Rhythmic Delta Activity) Focal or diffuse slow waves Rhythmic delta waves, diffuse or anterior predominance, extreme delta-brush Triphasic slow complexes, diffuse or anterior predominance Periodic activities: lateralized, bilateral asynchronous, generalized Interictal epileptic abnormalities: spikes, slow spikes, polyspikes, spike and wave, polyspike and wave Recording of a focal or generalized seizure or a focal or generalized status epilepticus Burst-suppression, suppression	

Etiology	Background activity	Superimposed abnormalities	Remarks
Sepsis-associated encephalopathy	Theta or delta slowing Sometimes suppression	Triphasic waves	Absence of reactivity in severe cases
Metabolic or toxic encephalopathy	Theta or delta slowing Sometimes suppression	Triphasic slow complexes, with anterior predominance, preserved reactivity	When present, preserved reactivity helps rule out status epilepticus Reactivity may be absent in severe cases
Infectious encephalitis (Herpetic)	Theta or delta slowing	Temporal focal slowing followed by focal periodic activities between Day 2 and Day 6, anterior and often temporal, unilateral, and with large amplitude, prolonged duration (1–1.5 s), periodic repetition with a short period (< 4 s)	Reactivity may be absent in severe cases
Autoimmune and paraneoplastic encephalitis	Theta or delta slowing	Periodic activities and more specific anomalies such as extreme delta-brush (delta activities overlaid with fast rhythms, bilateral, symmetrical, synchronous, with rhythmic repetition)	Variable EEG reactivity
Posterior reversible encephalopathy syndrome (PRES)	Theta or delta slowing	Slow waves, sometimes focal slow spikes with a posterior distribution	Variable EEG reactivity

EXPERT OPINION

*Rationale* Patients with SAE may develop seizures or status epilepticus (with or without the clinical correlation), which are frequently refractory to antiseizure medication and contribute to a poor prognosis (around 30% in cases of infectious or autoimmune etiologies, [22–24]; 70–80% for PRES [25]). A 20-min video-EEG with at least 8 electrodes should, when possible, be performed urgently, to detect nonconvulsive seizures. Nonconvulsive seizures and status epilepticus can be difficult to diagnose in patients with SAE. In the absence of rhythmic repetition and changes in frequency and location, the presence of sporadic epileptiform discharges (such as spike or spike-waves) does not indicate a seizure [26]. The presence of EEG reactivity to stimulation (i.e. any change in cerebral EEG activity following stimulation, excluding artifacts) is a simple clinical test to assess on EEG, and helps to rule out epileptic activity. EEG may reveal nonspecific abnormalities (for instance slower EEG background activity, frontal intermittent rhythmic discharges) or abnormalities that may guide the etiological diagnosis of severe acute encephalopathy. Periodic discharges are common but are not specific of a given etiology. Their temporal localization may suggest herpetic encephalitis (30% of patients) [27]. Generalized Periodic Discharges with triphasic morphology are in favor of metabolic or toxic encephalopathies, rhythmic delta activities, with extreme delta brush, point to anti-NMDA encephalitis [28, 29].

***Question 1.4: In a patient with severe acute encephalopathy, what urgent laboratory tests should be conducted for etiological purposes?***

**R 1.4.1: Experts suggest routinely measuring capillary blood glucose, verified with a blood sample, in the presence of SAE, to exclude hypoglycemia.**

EXPERT OPINION

**R 1.4.2: Experts suggest that laboratory tests requested in the context of SAE be performed sequentially based on historical elements and epidemiological data (**Table 4**).**

**Table 4:** Biological investigations in a patient presenting with severe acute encephalopathy

Situations	Samples
**Systematically**	Capillary blood glucose (controlled with venous blood glucose)
*As a first-line approach*	Complete blood count (CBC), platelets, blood electrolytes, liver function test, hemostasis, C-Reactive Protein (CRP)
*As a second-line approach*	Arterial blood gas, calcium level, phosphorus level
*As a third-line approach*	Ammonia level
*As a fourth-line approach*	Cortisol, TSH (Thyroid Stimulating Hormone), HBV (Hepatitis B Virus) serology, HCV (Hepatitis C Virus) serology, HIV serology, syphilitic serology
**Specific circumstances**	
Chronic kidney disease	Urea, creatinine, drugs blood concentration
Cirrhosis, Chronic Liver Disease	Ammonia level, sodium level, Prothrombin Time (factor V), platelet count
Chronic respiratory disease	PCO_2_
Abuse of Legal or Illicit Substances	Urinary and blood toxicology screening
Risk of vitamin deficiency	Vitamin B12, Folate, B1, PP, C levels
Immunodepression	Microbiological cultures, blood leukocytes, CRP
Return from a tropical country	Thickdrop and bloodsmear evaluation
Fever	Microbiological samples, blood leukocytes, CRP
Pregnant woman	Hepatic enzymes, platelet count
Endocrine Disorder	Cortisol, TSH
Autoimmune Predisposition or Background	Electrophoresis and immunoelectrophoresis of serum proteins, anti-DNA antibodies, antinuclear antibodies, (antineuronal antibodies)
Neoplasm	Calcium level, antineuronal antibodies
Family disease, consanguinity	Ammonia level, lactate, pyruvate, homocysteine levels
Toxics	Carbon monoxide, lead level

EXPERT OPINION

*Rationale* Limited data are available on the usefullness of blood sampling for the etiological work-up of SAE. Anamnestic and epidemiological clues will be the most important for the diagnosis [30, 31]. Indication to a specific exploration depends on the frequency of the cause and the potential consequences of delayed treatment. Whatever the suspected diagnosis, capillary determination of blood glucose levels is valuable to rule out hypoglycemia. A diagnostic work-up is discussed in Table 4. In the absence of any obvious cause of severe acute encephalopathy, blood ammonemia should be measured due to its possible therapeutic implication if elevated.

***Question 1.5: In a patient with SAE, when should a lumbar puncture be performed for etiological purposes? What initial analyses should be requested based on suspected etiologies?***

**R 1.5.1: Experts suggest performing a lumbar puncture (in the absence of contraindications) in patients with SAE in the following cases: immunocompromised status; fever; meningeal syndrome; suspicion of encephalitis on imaging or EEG.**

EXPERT OPINION

**R 1.5.2: In a patient with SAE, when lumbar puncture is indicated, experts suggest conducting systematic first-line investigations and then second-line investigations in case of negative results of first-line examinations or specific elements suggesting an etiological orientation (**Table 5**).**

**Table 5:** Cerebrospinal fluid investigations in patients with severe acute encephalopathy

**First-line investigations** *Febrile SAE or with signs suggestive of CNS infection*
Measurement of opening pressure in the lying position Multiplex PCR panel for "meningitis/encephalitis" (1) OR PCR for HSV-1, HSV-2, VZV and enterovirus
Direct bacteriological examination of cerebrospinal fluid (CSF) with cell quantification, leukocyte formula, and Gram staining
Bacterial culture
Protein analysis in CSF Glucose level in CSF and serum glucose level
**Second-line investigations** *Unexplained SAE with negative initial tests (non-exhaustive list)*
Antineuronal Antibodies (combined blood and cerebrospinal fluid tests)
Intrathecal synthesis of Ig (oligoclonal bands)
High-throughput sequencing "NGS" for infectious agents
Venereal Disease Research Laboratory (VDRL) test
*Mycobacterium tuberculosis* (PCR, direct examination, and specific cultures) if not performed initially
**If immunosuppression**
PCR for CMV, EBV, HHV6-7
HIV PCR if known HIV infection, to be correlated with serum viral load
Mycological examination, including at least Cryptococcus search (India ink, specific culture, and antigen testing)
*Toxoplasma gondii* PCR
*Mycobacterium tuberculosis* (PCR, direct examination, and specific cultures)
JC virus PCR
Hepatitis E virus
**If there is a history of travel to an endemic area** *(to be adjusted based on epidemic context and clinical presentation or exposure to risk)*
Arboviruses (West Nile virus, dengue, Zika, chikungunya, Japanese encephalitis, tick-borne encephalitis, Nipah virus)
Rabies testing (simultaneously on cerebrospinal fluid, saliva, and skin biopsy)
Trypanosomiasis testing
Histoplasmosis testing (PCR on blood and CSF)
Leptospirosis testing (PCR on blood and CSF)

(1)Several kits are in development

EXPERT OPINION

*Rationale* In SAE patients, the diagnostic yield of lumbar puncture (LP) is estimated between 10 and 30%. In patients with any signs suggestive of CNS infection, LP should be widely performed considering (i) the poor negative predictive value of classic clinical signs (including neck stiffness) for ruling out infectious meningitis or encephalitis, (ii) the significant prognostic impact of treatment delay in infectious encephalitis, and (iii) the low morbidity of LP (rate of severe complications < 0.5%) [32]. When LP is performed, the first line tests should focus on identifying common infectious etiologies that require specific treatment: pyogenic bacteria, HSV, VZV, and *Mycobacterium tuberculosis* for all patients, and other etiologies depending on associated factors (Table 5). The use of multiplex CSF PCR warrants cautious interpretation. Systematic reviews report false negative rates as high as 24.5% and 9.6% for HSV-1 and VZV infections, respectively [33]. In contrast, specificity appears high for both bacterial and viral pathogens. False negative LPs have been reported in 4% of patients with HSV encephalitis, exclusively in CSF sampled less than 4 days after symptom onset [34]. Therefore, repeat, or extended investigations to rule out HSV encephalitis should best be performed on a second CSF analysis sampled at least 4 days after symptom onset.

***Question 1.6*** ***:*** ***In a patient with SAE, when should cerebral imaging be performed before a lumbar puncture to reduce the risk of complications?***

**R 1.6: Experts suggest performing cerebral imaging before lumbar puncture to reduce the risk of complications in the presence of focal neurological deficits and/or signs of brain herniation and/or seizures.**

EXPERT OPINION

*Rationale* There are no interventional studies indicating that pre- LP imaging reduces complications in SAE. In a retrospective study involving 64 patients where brain imaging was considered before LP, a normal clinical examination had a negative predictive value of 0.85 (0.73–0.97) for assessing the reliability of a normal clinical examination to rule out the need for a CT scan. [35]. In the largest cohort study of adult patients with proven acute bacterial meningitis, 47/1533 patients (3%) deteriorated (altered consciousness or cardiorespiratory failure) within 8 h after LP, and only two (0.1%) deteriorated within one hour after LP [36]. In a multi-center retrospective study of 202 patients with acute bacterial meningitis, the comparison of international guidelines (American, English, European, and Swedish) to assess the diagnostic value of cranial imaging before LP showed that only American recommendations did not miss major intracranial abnormalities or findings requiring neurosurgical intervention [37]. However, a prospective cohort of 815 Swedish patients demonstrated reduced mortality and increased favorable outcomes with adherence to Swedish recommendations regarding neuroimaging indications before LP (versus European and American recommandations). The authors concluded that altered mental status and immunocompromised status should not represent indications per se to peform imaging before LP [38]. French recommendations for community-acquired bacterial meningitis limit pre-LP imaging indications to signs suggesting intracranial processes, cerebral herniation, and persistent convulsive seizures, offering a compromise between urgent diagnosis and patient safety [39].

**Field 2: Indications and methods of neuromonitoring**

***Question 2.1: In a patient with SAE, should clinical scores be used for monitoring and adjusting management?***

**R 2.1.1: In a patient with SAE, appropriate scores for delirium (CAM-ICU or ICDSC) or coma (GCS or FOUR score) monitoring must be used in order to tailor diagnostic and therapeutic management.**

GRADE 1 + / STRONG AGREEMENT.

**R 2.1.2: In a patient with SAE secondary to alcohol withdrawal syndrome, specific scores for monitoring and adapting therapeutic management should probably be used.**

GRADE 2 + / STRONG AGREEMENT.

**R 2.1.3: Experts suggest not limiting monitoring of patients with SAE solely to the use of clinical scores for adjusting diagnostic and therapeutic management.**

EXPERT OPINION

**R 2.1.4: In a patient with SAE clinically manifesting as prolonged coma, experts suggest using the Coma Recovery Scale-Revised (CRS-R) to track any changes in consciousness.**

EXPERT OPINION

*Rationale* Neurologic scores serve as indispensable tools in the ICU to objectively assess and monitor neurological function in critically ill patients. These scores, such as the Glasgow Coma Scale (GCS) [3] and the FOUR score [4], provide a standardized framework for bedside assessment of impairment of the level of consciousness. The FOUR score provides greater neurological detail than the GCS, recognizes a locked-in syndrome, and is superior to the GCS due to the availability of brainstem reflexes, breathing patterns, and the ability to recognize different stages of herniation. Other scores, such as the CAM-ICU [40] and the Intensive Care Delirium Screening Checklist (ICDSC) [41] have been developed for monitoring of delirium during ICU stay. In the ICU, these scores enable clinicians to promptly identify changes in neurological status, guiding treatment decisions and facilitating communication among multidisciplinary teams. The Coma Recovery Scale-Revised (CRS-R) has been recommended in numerous international guidelines for the assessment of persistent coma [42]. Several studies showed clinical benefit when specific scales, such as the clinical institute withdrawal assessment [43] or the modified Minnesota detoxification scales were used in the management of the alcohol withdrawal syndrome [44].

***Question 2.2: In a patient with SAE, can clinical scores be used by the paramedical team to enhance monitoring?***

**R 2.2: In a patient with SAE, experts propose that clinical scores be used by the paramedical team after being trained to enhance monitoring.**

EXPERT OPINION

*Rationale* Clinical scores play a crucial role in enhancing neuromonitoring within the ICU, empowering paramedical teams with standardized tools to assess and track neurological function [41, 42, 45]. By utilizing these scores, paramedics can efficiently evaluate patients' neurological status at the bedside. These scores provide a structured framework for communication between paramedical staff and other healthcare professionals, facilitating seamless collaboration and ensuring consistent monitoring of neurological changes over time.

***Question 2.3: In a patient with SAE, should transcranial doppler be used to tailor management?***

**R 2.3: Experts suggest considering performing transcranial doppler to detect intracranial hypertension in patients with SAE, as in patients with brain injury, in conjunction with other diagnostic tools, especially imaging.**

EXPERT OPINION

*Rationale* Transcranial Doppler (TCD) is a diagnostic tool used to assess cerebral blood flow velocity (CBFV) in major intracranial vessels. In critical care settings, this measurement is often employed for non-invasive estimation of intracranial pressure (ICP) or detection of cerebral vasospasm [46]. Additionally, continuous monitoring of CBFV and blood pressure allows for the calculation of the mean flow velocity index (Mxa), which is valuable for quantifying cerebral autoregulation [47]. In patients with acute encephalopathy of various etiologies, abnormalities in CBFV (e.g., decreased mean CBFV, mean flow velocity, suggesting reduced cerebral blood flow; reduced diastolic CBFV, FVd, or increased pulsatility index, indicating elevated ICP and/or impaired cerebral autoregulation (e.g., Mxa > 0.3) have been identified [48]. These abnormalities are associated with increased mortality and unfavourable neurological outcomes. However, the role of TCD in guiding patient management remains uncertain, as well-defined pathological threshold values (e.g., diastolic flow velocity < 20 cm/sec or PI > 1.2) triggering interventions are lacking, and the effects of different therapies on TCD and cerebral function have been inadequately studied [49–51].

***Question 2.4: In a patient with SAE, should EEG (intermittent or continuous) be used to tailor management?***

**R 2.4.1: In a patient with SAE, experts suggest performing EEG monitoring in the absence of rapidly favorable clinical evolution to investigate an uncontrolled or superimposed factor of cerebral aggression.**

EXPERT OPINION

**R 2.4.2: In patients with SAE complicated by coma and/or secondary to an inflammatory or infectious cause, experts suggest preferably conducting continuous video EEG (24 to 72 h) rather than standard EEG to rule out the presence of non-convulsive seizures or non-convulsive status epilepticus.**

EXPERT OPINION

*Rationale* Among patients with altered consciousness in ICU, 10–60% experience epileptic seizures [52, 53], which are predominantly nonconvulsive in about 80% of cases. These seizures are most often (80%) detected within the first 24 h following admission, but 20% of the comatose only experience seizures after the first 24 h [54–58]. The presence of non-convulsive seizures is associated with a twofold higher mortality rate [56, 57].

In patients at high risk of epileptic complications (comatose patients and/or those for whom an infectious cause of encephalopathy is suspected), prolonged video-EEG monitoring (24–72 h) is therefore probably preferable to standard EEG) [59]. The EEG monitoring should include at least 8 electrodes and be systematically associated with video recording (video-EEG monitor) to allow quality review. The intensive care staff must be trained to operate the device (starting, repositioning of electrodes, inserting notes, repositioning of the video) so that the recording remains of good quality even outside the opening hours of the neurophysiology laboratory. Intensivists and nurses can be trained to recognize certain common EEG patterns (trace composed of sharp generalized rhythmic figures < 2.5 Hz non-reactive, suggestive of epilepsy vs slower figures possibly triphasic pseudo-rhythmic > 2.5 Hz reactive, suggestive of toxic/medicinal encephalopathy) with the aid, if possible, of quantified analysis (amplitude, spectral power) facilitating the quick review of long recordings [60]. Daily interaction between neurophysiologists and intensivists is strongly recommended given the difficulty of interpreting ICU EEGs [59, 61].

***Question 2.5: In a patient with SAE, should intracranial pressure monitoring be used to improve prognosis?***

**R 2.5: In patients with SAE, experts propose not routinely performing invasive intracranial pressure monitoring. A discussion with an expert neurocritical care center to assess the indication for intracranial pressure monitoring may be proposed on a case-by-case basis, especially in the most severe patients, particularly those showing indirect signs of intracranial hypertension on imaging.**

EXPERT OPINION

*Rationale* In patients with SAE, the pathophysiology of acute encephalopathy may not primarily involve intracranial hypertension, therefore the utility of intracranial pressure monitoring depends on etiology and may be limited. The management of SAE patients guided by intracranial pressure monitoring has not been evaluated in a randomized controlled trial.

Among medical conditions more commonly associated with cerebral oedema and elevated ICP, like acute liver failure [62, 63] and meningitis, evidence of interventions tailored by ICP monitoring remain scarce and are mainly based on cases reports or small cohorts [64, 65]. ICP monitoring may provide valuable insights into intracranial dynamics in selected patients.

***Question 2.6: In a patient with SAE, should cerebral oximetry monitoring be used to improve prognosis?***

**R 2.6: Experts suggest not using cerebral oximetry monitoring for the initial management of patients with SAE.**

EXPERT OPINION

*Rationale* Evidence supporting the use of cerebral oximetry monitoring in patients with SAE is lacking. While cerebral oximetry offers a non-invasive method to monitor regional cerebral oxygen saturation, its efficacy and impact on patient outcomes in the specific context of acute encephalopathy remain unclear. Existing studies often involve heterogeneous patient populations with diverse etiologies of encephalopathy, making it challenging to draw definitive conclusions regarding the utility of cerebral oximetry in this setting.

***Question 2.7: In a patient with SAE, what are the preventive therapeutic tools for prevention of secondary insults of systemic origin to limit the occurrence of secondary lesions and improve prognosis?***

**R 2.7: In patients with SAE, experts suggest the monitoring of secondary insults of systemic origin to limit the occurrence of secondary brain lesions and improve prognosis.**

EXPERT OPINION

*Rationale* Secondary insults of systemic origin are a heterogeneous group of factors that can exacerbate primary brain injury. In patients with SAE, temperature is the most studied factor evaluated in epidemiological studies of various types of CNS presentations, where both fever and hypothermia were shown to be associated with poor outcomes [66–68]. The relationship between peak temperature in the first 24 h after ICU admission and in-hospital mortality differs between traumatic brain injury/stroke and CNS infection. For CNS infection, increased temperature is not associated with increased risk of death [69], and can probably be tolerated in the absence of worsening of consciousness [70]. In a multicenter study, systemic secondary brain insults were not associated with outcome in critically ill patients with convulsive status epilepticus [71]. Interventional studies targeting hypothermia failed to show neuroprotection in patients with status epilepticus requiring mechanical ventilation [72] or acute liver failure [73], and were even associated with increased mortality in patients with severe community-acquired infection [74].

**FIELD 3: Prognostication of awakening and neurologic sequelae**

***Question 3.1: In a patient with SAE, are clinical scores useful for the assessment of neurological prognosis? If yes, which ones?***

**R 3.1.1: In patients with SAE, clinically manifested as delirium, at least daily CAM-ICU (to quantify its duration and qualify its phenotype) and sedation scale (RASS) monitoring should be performed to assess the vital and cognitive prognosis of a delirium episode.**

GRADE 2 + / STRONG AGREEMENT.

**R 3.1.2: In patients with SAE, clinically manifested as a coma, using the FOUR score (and the BRASS score in sedated patients) should be preferentially used rather than the Glasgow Coma Scale to assess the depth of coma and brainstem responses.**

GRADE 2 + / STRONG AGREEMENT.

**R 3.1.3: In a patient with SAE, manifested as persistent impaired consciousness (without clearly defined duration), the Coma Recovery Scale-Revised (CRS-R) for the diagnosis and prognosis of consciousness recovery, as well as for functional prognosis, should be probably used.**

GRADE 2 + / STRONG AGREEMENT.

**R 3.1.4: In patients with SAE related to hepatic encephalopathy or autoimmune encephalitis, a specific score (West-Haven Score and CASE score, respectively) should be used to assess the prognosis.**

GRADE 2 + / STRONG AGREEMENT.

*Rationale* The neurological outcome of patients is globally related to the burden of AE during the ICU stay and several severity scores have been associated with both the vital and functional prognosis. In delirious patients, duration [75], motoric subtype (notably hypoactive and mixed subtypes) [76], and severity of delirium [77], all assessed by the CAM-ICU-7 combined with the RASS, have been associated with either long-term cognitive impairment or mortality. In comatose patients within 48 h of ICU admission, FOUR score to assess coma depth and brainstem response has demonstrated a slightly better association with the mortality and 3-month functional outcome than the Glasgow Coma Score, which is also acceptable [78, 79]. There are no data on prognostic performances of both scores in the later phase of critical illness. Assessment of brainstem responses in deeply sedated patients, using the Brainstem Response Assessment Sedation Scale (BRASS), has also proved interesting in predicting day 28 occurrence of delirium and mortality [80, 81]. During the subacute and chronic phase, recovery of consciousness is better assessed by the CR [42], with worse functional prognosis being associated with the severity of consciousness impairment [82]. Lastly, in some etiologies, specific severity scores are independently associated with patient’s outcome, such as the West-Haven for mortality in hepatic encephalopathy [83] and the Clinical Assessment Scale in Autoimmune Encephalitis (CASE) [84] or the anti-NMDAR Encephalitis One-Year Functional Status (NEOS) score [85] for functional outcome in autoimmune encephalitis [86].

***Question 3.2: In a patient with SAE, should automated pupillometry be used to assess prognosis? If yes, in which situation(s)?***

**R 3.2: In a patient with SAE, experts suggest not using automated pupillometry systematically to assess prognosis.**

EXPERT OPINION

*Rationale* Monocentric studies provide low-level evidence for the use of automated pupillometry to predict the occurrence of acute encephalopathy in the ICU [87, 88]. Of note, none of them focused on long-term prognosis.

Single-center studies evaluated automated pupillometry for prediction of mortality in patients admitted for sepsis [89], in patients on veno-arterial extracorporeal membrane oxygenation for refractory cardiogenic shock [90]**,** in hepatic encephalopathy [91], and after liver transplantation [92]. There are published data on correlations between automated pupillometry parameters and EEG patterns to characterize the severity of acute encephalopathy [93, 94].

***Question 3.3: In a patient with SAE, which imaging studies (CT scan, MRI, PET scan) should be used to assess prognosis?***

**R 3.3.1: In a patient with SAE outside of specific etiologies (see R 3.3.2), magnetic resonance imaging should not be systematically used to assess prognosis.**

GRADE 2-/ STRONG AGREEMENT

**R 3.3.2: In a patient with SAE secondary to infectious or autoimmune encephalitis, or PRES, brain MRI should probably be used to assess prognosis.**

GRADE 2 + / STRONG AGREEMENT.

*Rationale* No high-quality study has successfully established a correlation between brain imaging patterns and prognosis in SAE [15, 95]; encompassing all-cause encephalitis [96]. Only brain oedema has been linked to unfavourable outcomes in all-cause encephalitis [97]. In the context of sepsis-associated encephalopathy, the presence of MRI abnormalities, such as strokes and leukoaraiosis, may be linked to a more adverse prognosis in terms of survival or functional outcomes [16, 98–100]. In the context of *Herpes simplex virus* encephalitis, multiple studies have underscored the correlation between the extent of lesions in brain MRI, restricted diffusion and long-term functional prognosis [68, 101, 102]. Specifically, FLAIR hyperintensity spanning over three lobes, bilateral diffusion enhancement, and thalamic involvement have been associated with poor functional outcomes, especially in elderly patients [101]. In Varicella-Zoster Virus encephalitis, a connection has been observed between vasculitis diagnosed on MRI and functional prognosis [103–105]. Brain imaging prognosis value for anti-NMDA receptor (NMDAR) encephalitis is debated [106–108]. Nevertheless, in severe cases of all-cause autoimmune encephalitis and anti-NMDAR encephalitis, normal MRI results can be considered a promising prognostic marker [85, 109], whereas hippocampus involvement as an unfavourable marker [110]. Data is lacking to associate any specific pattern with poor outcomes in the 90% of patients with abnormal MRI in Acute Disseminated Encephalomyelitis (ADEM) admitted to the ICU [111, 112]. In PRES, the presence of subarachnoid or intraparenchymal haemorrhage, has been linked to mortality or persistent disability [113–116]. The prognostic significance of restricted diffusion or gadolinium enhancement remains contentious [113, 114]. Knowledge about brain imaging and metabolic (i.e. hyperuremic, hepatic, hypoglycaemic) encephalopathy prognosis is scarce. To date, PET imaging has not yet been evaluated as a prognostic marker in any SAE cohort study.

***Question 3.4: In a patient with SAE, which electrophysiological examination(s) (EEG, evoked potentials,*** **etc** ***.) should be used to assess prognosis?***

**R 3.4: Experts suggest that an EEG should be routinely performed to assess the vital and functional prognosis in the presence of severe acute encephalopathy.**

EXPERT OPINION

*Rationale* The analysis of basic EEG parameters such as dominant frequency, amplitude, continuity, and reactivity, as well as the description of any paroxysms that may appear on this background activity, provide crucial diagnostic and prognostic information in intensive care settings [55, 56]. Lateralized periodic discharges (LPDS) are primarily associated with brain injuries (i.e. strokes, *Herpes simplex* encephalitis). Periodic discharges or slow waves with triphasic morphology and intermittent rhythmic delta activities in the frontal region (FIRDA) typically indicate metabolic or toxic disturbances [117, 118]. EEG changes associated with the depth of coma can be summarized as follows: initially, EEG rhythms gradually slow towards lower frequencies and amplitude increases. Subsequently, amplitude begins to decrease, reactivity disappears, and the EEG signal becomes first discontinuous and ultimately disappears, resulting in a flat EEG trace or electrocerebral silence (suppression from the entirety of the record). The lack of EEG reactivity is strongly associated with mortality [117, 118].

Evoked potentials (EPs) are quite complementary to EEG [119]. Somatosensory evoked potentials (SEPs) assess the functionality of the somatosensory system from the stimulated peripheral nerve to the primary sensory cortex (S1 area), via the posterior columns of the spinal cord and the brainstem (lemniscal pathway). Early auditory evoked potentials also named brainstem auditory evoked potentials (BAEPs) track auditory impulses from the inner ear through the brainstem. Middle latency auditory evoked potentials (MLAEPs) assess post-synaptic activity in mesodiencephalic auditory relays and the primary auditory cortex. Long latency auditory evoked potentials also known as auditory event related potentials (ERPs) examine cortical areas involved in cognitive processes [120]. EPs detect nerve pathway impairments caused by various neuronal injury mechanisms. Slowed conduction time may stem from demyelination, while significant decrease in amplitude or absent responses could indicate axonal injury in brain dysfunctions. Moderate and reversible EP alterations suggest possible recovery or mild sequelae, whereas severe alterations or cortical response loss are associated with poor outcomes [121].

***Question 3.5: In a patient with SAE, which blood biomarker(s) should be measured to assess prognosis?***

**R 3.5: In a patient with SAE, blood biomarkers (i.e., ammonia, neuron-specific enolase, protein S100b, Neuro-Filament Light, Brain-derived neurotrophic factor, N-Terminal pro C-Type Natriuretic Peptide pro and anti-inflammatory cytokines, CRP, and PCT) should not be measured to assess long-term vital or neurological functional prognosis.**

GRADE 2-/ STRONG AGREEMENT

*Rationale* In patients with cirrhosis and hepatic encephalopathy (HE), studies note a correlation between HE severity and blood ammonia levels [122–125]. Establishing a discriminating threshold is challenging, and some studies don’t find this correlation [126–128]. The positive predictive value of high ammonia levels for HE diagnosis is modest [128]. Thus, ammonia measurement isn't recommended to confirm HE diagnosis or assess its severity, as some HE-free patients may have high ammonia levels. Conversely, the negative predictive value is interesting, excluding HE if ammonia is < 30 µmol/L [122, 124, 127]. The ammonia level is relevant in cirrhotic patients only if the etiology of encephalopathyremains doubtful. Limited data exists on the correlation between ammonia kinetics and HE evolution [122, 125]. Some studies link high ammonia levels to ICU mortality [125, 129]. However, the absence of a robust threshold and the lack of correlation in other studies [126, 130] suggest not using ammonia levels to predict mortality.

Some studies assess biomarkers' prognostic value in different encephalopathy etiologies (hepatic, sepsis-related, and carbon monoxide intoxication). Biomarkers of brain cellular damage include S100beta protein, Neuron-Specific Enolase (NSE), Neuro-filament Light (NFL), Brain-derived neurotrophic factor (BDNF), and N-Terminal pro C-Type Natriuretic Peptide (NTproCNP). Different outcomes were evaluated, including initial encephalopathy severity, evolution, death occurrence, delirium, organ failure in ICU, and long-term psycho-cognitive disabilities [79, 131–140]. Limited sample size, absence of external validation and discordant results among studies suggest that blood biomarkers of brain injury should not be used for prognostic assessment of patients with acute and severe ICU encephalopathy, regardless of etiology.

**FIELD 4: Management (Excluding etiological treatment)**

***Question 4.1: In a patient with SAE, should certain medications be avoided to prevent worsening of the neurological status?***

**R 4.1.1: In a patient presenting with SAE, experts suggest, when multiple options are available, prioritizing drugs less frequently associated with neurological toxicity, having the highest therapeutic index, and the shortest half-life.**

EXPERT OPINION

**R 4.1.2: In a patient presenting with SAE, experts suggest adjusting drug doses according to the presence of renal and/or hepatic failure and monitoring plasma concentrations of neurotoxic drugs.**

EXPERT OPINION

**R 4.1.3: In a patient presenting with SAE, experts suggest not prescribing nefopam or tramadol in patients requiring level 2 analgesics.**

EXPERT OPINION

**R 4.1.4: In a patient presenting with SAE requiring sedation during mechanical ventilation, experts suggest prioritizing drugs other than benzodiazepines (outside specific indications) to prevent delirium and delayed awakening.**

EXPERT OPINION

*Rationale* There are currently no studies assessing the impact of prescribed medications on the deterioration of neurological conditions in patients with severe acute encephalopathy (SAE). Nevertheless, among the drugs commonly administered in intensive care units, some are associated with a heightened risk of delirium, while alternative options can be considered. Nefopam may contribute to delirium even at therapeutic doses [141], and tramadol is associated with a greater risk of postoperative delirium compared to other opioids [142]. The use of benzodiazepines for sedating mechanically ventilated patients is associated with a higher incidence of delirium compared to propofol or dexmedetomidine [143, 144]. In a broader context, when dealing with a therapeutic class that is likely to exacerbate the neurological state of patients with SAE, it may be important to prioritise medicines with the highest therapeutic index and shortest half-life, in order to ensure an optimal safety profile [145]. Monitoring plasma drug concentrations could prove beneficial in preventing neurotoxicity, particularly in cases where neurotoxicity is dose-dependent or when there is liver or kidney failure, both of which can affect drug pharmacokinetics [146].

***Question 4.2: In a patient with SAE, should non-pharmacological measures be implemented to improve neurological status?***

**R 4.2: In patients with severe SAE clinically manifested by delirium, experts suggest using the 'ABCDEF' bundle to reduce the delirium burden.**

EXPERT OPINION

*Rationale* The ABCDEF bundle is a multifaceted approach designed to improve patient outcomes and reduce the incidence of delirium in the ICU (Table 6) [2]. In large multicenter observational studies, the use of the ABCDEF bundle in ICU patients showed significant and clinically meaningful improvements in outcomes including survival, mechanical ventilation use, coma, delirium, restraint-free care, ICU readmissions, and post-ICU discharge disposition [147]. Although randomized clinical studies are lacking, this multifaceted approach is recommended to reduce delirium burden in ICU.

**Table 6:** The ABCDEF bundle

**A**ssess, Prevent, and Manage Pain
**B**oth Spontaneous Awakening Trials (SAT) and Spontaneous Breathing Trials (SBT)
**C**hoice of analgesia and sedation
**D**elirium: Assess, Prevent, and Manage
**E**arly mobility and Exercise
**F**amily engagement and empowerment

## Data Availability

Data supporting this paper will be made available upon request to the corresponding author.
